# Supplementary material for: Effects of Chlorogenic Acid on Performance, Anticoccidial Indicators, Immunity, Antioxidant Status, and Intestinal Barrier Function in Coccidia-Infected Broilers
Source: Animals (Basel). 2022 Apr 8;12(8):963. doi: 10.3390/ani12080963 (PMC9030001; doi:10.3390/ani12080963)
Supplement: Supplementary file 1 [file animals-12-00963-s001.zip › animals-1646745-supplementary.pdf]

# Supplementary Materials

# File S1

**D-lactic acid, DAO and Immunity Indicators ELISA Kit Instructions**

| Index | Assay range | Sensitivity | $R^2$ | Kit No. |
|---|---|---|---|---|
| D-lactic acid | 2.5 nmol/L – 80 nmol/L | less than 1.0 nmol/L | 0.9930 | YJ51407 |
| DAO | 0.75 U/mL - 24 U/mL | less than 0.1 U/mL | 0.9969 | ML036981 |
| IL-6 | 1 pg/mL – 32 pg/mL | less than 0.1 pg/mL | 0.9976 | ML042757 |
| IL-10 | 2.5 pg/mL – 80 pg/mL | less than 0.1 pg/mL | 0.9988 | ML059830 |
| TNF-α | 2.5 pg/mL - 80 pg/mL | less than 0.1 pg/mL | 0.9990 | ML002790 |
| IgA | 10 μg/mL - 320 μg/mL | less than 1.0 μg/mL | 0.9979 | ML002792 |

# D-Lactic acid (D-LA) ELISA Kit Instructions (YJ51407)

## 1. Sample collection and storages

Use a serum separator tube and allow samples to clot for 30 minutes before centrifugation for 10 minutes at approximately 3000×g. Remove serum and assay immediately or aliquot and store samples at -20°C or -80°C.Avoid repeated freeze-thaw cycles

## 2. Assay procedure

1. Prepare all reagents before starting assay procedure. It is recommended that all Standards and Samples be added in duplicate to the Microelisa Stripplate.
2. Add standard: Set Standard wells, testing sample wells. Add standard 50 µl to standard well.
3. Add Sample: Add testing sample 10 µl then add 40 µl of Sample Diluent to testing sample well; Blank well doesn't add anything.
4. Add 100 µl of HRP-conjugate reagent to each well, cover with an adhesive strip and incubate for 60 minutes at 37°C.
5. Aspirate each well and wash, repeating the process four times for a total of five washes. Wash by filling each well with Wash Solution (400 µl) using a squirt bottle, manifold dispenser or autowasher. Complete removal of liquid at each step is essential to good performance. After the last wash, remove any remaining Wash Solution by aspirating or decanting. Invert the plate and blot it against clean paper towels.
6. Add chromogen solution A 50 µl and chromogen solution B 50 µl to each well. Gently mix and incubate for 15 minutes at 37°C. Protect from light.
7. Add 50 µl Stop Solution to each well. The color in the wells should change from blue to yellow. If the color in the wells is green or the color change does not
appear uniform, gently tap the plate to ensure thorough mixing.
8. Read the Optical Density (O.D.) at 450 nm using a microtiter plate reader within 15 minutes

## 3. Calculate

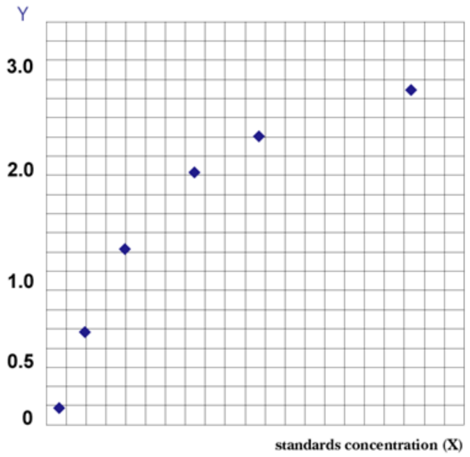


## 4. Assay range

2.5 nmol/L – 80 nmol/L

## 5. Sensitivity

The minimum detectable dose is typically less than 1.0 U/mL.

**Diamine oxidase (DAO) ELISA Kit Instructions (ML036981)**

**1. Sample collection and storages**

Coagulation at room temperature 10-20 mins, centrifugation 20 mins at the speed of 2000-3000 r. p. m. remove supernatant, if precipitation appeared, Centrifugal again.

**2. Assay procedure**

1. Add standard: Set Standard wells, testing sample wells. Add standard 50 µl to standard well.
2. Add sample: Set blank wells separately (blank comparison wells don't add sample and HRP-Conjugate reagent, other each step operation is same). testing sample well. add Sample dilution 40 µl to testing sample well, then add testing sample 10 µl (sample final dilution is 5-fold), add sample to wells, don't touch the well wall as far as possible, and gently mix.
3. Incubate: After closing plate with Closure plate membrane, incubate for 30 min at 37℃.
4. Configurate liquid: 30-fold (or 20-fold) wash solution diluted 30-fold (or 20-fold) with distilled water and reserve.
5. Washing: Uncover Closure plate membrane, discard Liquid, dry by swing, add washing buffer to every well, still for 30 s then drain, repeat 5 times, dry by pat.
6. Add enzyme: Add HRP-Conjugate reagent 50 µl to each well, except blank well.
7. Incubate: Operation with 3.
8. Washing: Operation with 5.
9. Color: Add Chromogen Solution A 50 ul and Chromogen Solution B to each well, evade the light preservation for 15 min at 37℃
10. Stop the reaction: Add Stop Solution50µl to each well, Stop the reaction (the blue color change to yellow color).
11. Assay: take blank well as zero, read absorbance at 450 nm after Adding Stop Solution and within 15 min.

**3. Calculate**

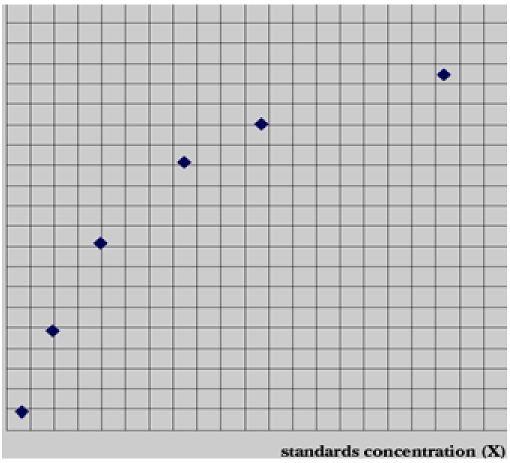


**4. Assay range**

0.75 U/mL - 24 U/mL.

**5. Sensitivity**

The minimum detectable dose is typically less than 0.1 U/mL.

**Interleukin 6 (IL-6) ELISA Kit Instructions (ML042757)**

**1. Sample collection and storages**

Coagulation at room temperature 10-20 mins, centrifugation 20 mins at the speed of 2000-3000 r. p. m. remove supernatant, if precipitation appeared, Centrifugal again.

**2. Assay procedure**

1. Add standard: Set Standard wells, testing sample wells. Add standard 50 μl to standard well.
2. Add sample: Set blank wells separately (blank comparison wells don't add sample and HRP-Conjugate reagent, other each step operation is same). testing sample well. add Sample dilution 40 μl to testing sample well, then add testing sample 10 μl (sample final dilution is 5-fold), add sample to wells, don't touch the well wall as far as possible, and gently mix.
3. Add enzyme: Add HRP-Conjugate reagent 100 μl to each well, except blank well.
4. Incubate: After closing plate with Closure plate membrane, incubate for 60 min at 37°C.
5. Configurate liquid: 20-fold wash solution diluted 20-fold with distilled water and reserve.
6. Washing: Uncover Closure plate membrane, discard Liquid, dry by swing, add washing buffer to every well, still for 30 s then drain, repeat 5 times, dry by pat.
7. Color: Add Chromogen Solution A 50 μl and Chromogen Solution B to each well, evade the light preservation for 15 min at 37°C
8. Stop the reaction: Add Stop Solution 50 μl to each well, Stop the reaction (the blue color change to yellow color).
9. Assay: take blank well as zero, read absorbance at 450 nm after Adding Stop Solution and within 15min

**3. Calculate**

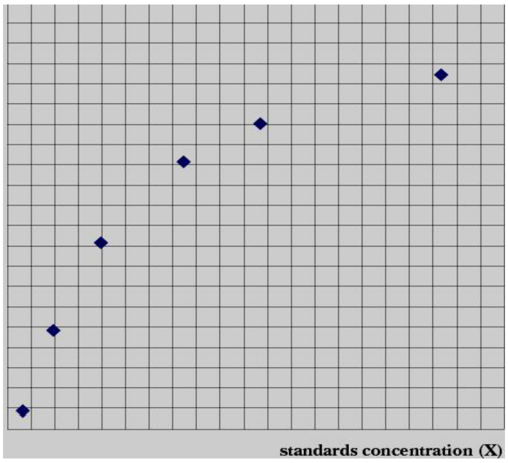


**4. Assay range**

1 pg/mL - 32 pg/mL.

**5. Sensitivity**

The minimum detectable dose is typically less than 0.1 pg/mL.

**Interleukin 10 (IL-10) ELISA Kit Instructions (ML059830)**

## 1. Sample collection and storages

Coagulation at room temperature 10-20 mins, centrifugation 20 mins at the speed of 2000-3000 r. p. m. remove supernatant, if precipitation appeared, Centrifugal again.

## 2. Assay procedure

1. Add standard: Set Standard wells, testing sample wells. Add standard 50 µl to standard well.
2. Add sample: Set blank wells separately (blank comparison wells don't add sample and HRP-Conjugate reagent, other each step operation is same). testing sample well. add Sample dilution 40 µl to testing sample well, then add testing sample 10 µl (sample final dilution is 5-fold), add sample to wells, don't touch the well wall as far as possible, and gently mix.
3. Add enzyme: Add HRP-Conjugate reagent 100 µl to each well, except blank well.
4. Incubate: After closing plate with Closure plate membrane, incubate for 60 min at 37°C.
5. Configurate liquid: 20-fold wash solution diluted 20-fold with distilled water and reserve.
6. Washing: Uncover Closure plate membrane, discard Liquid, dry by swing, add washing buffer to every well, still for 30s then drain, repeat 5 times, dry by pat.
7. Color: Add Chromogen Solution A 50 µl and Chromogen Solution B to each well, evade the light preservation for 15 min at 37°C
8. Stop the reaction: Add Stop Solution 50 µl to each well, Stop the reaction (the blue color change to yellow color).
9. Assay: take blank well as zero, read absorbance at 450 nm after Adding Stop Solution and within 15min.

## 3. Calculate

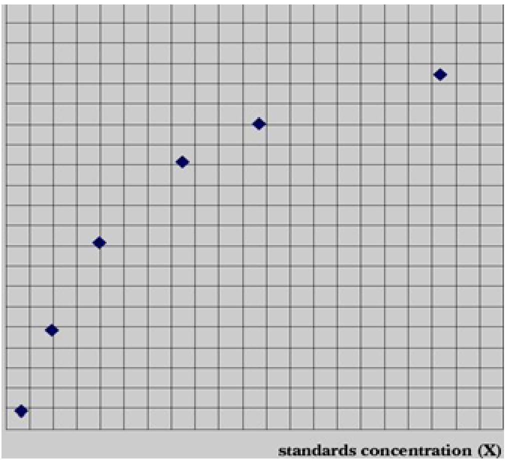


## 4. Assay range

2.5 pg/mL - 80 pg/mL.

## 5. Sensitivity

The minimum detectable dose is typically less than 0.1 pg/mL.

**Tumor necrosis factor α (TNF-α) ELISA Kit Instructions (ML002790)**

**1. Sample collection and storages**

Coagulation at room temperature 10-20 mins, centrifugation 20 mins at the speed of 2000-3000 r. p. m. remove supernatant, if precipitation appeared, Centrifugal again.

**2. Assay procedure**

1. Add standard: Set Standard wells, testing sample wells. Add standard 50 μl to standard well.
2. Add sample: Set blank wells separately (blank comparison wells don't add sample and HRP-Conjugate reagent, other each step operation is same). testing sample well. add Sample dilution 40 μl to testing sample well, then add testing sample 10 μl (sample final dilution is 5-fold), add sample to wells, don't touch the well wall as far as possible, and gently mix.
3. Incubate: After closing plate with Closure plate membrane, incubate for 30 min at 37°C.
4. Configurate liquid: 30-fold (or 20-fold) wash solution diluted 30-fold (or 20-fold) with distilled water and reserve.
5. Washing: Uncover Closure plate membrane, discard Liquid, dry by swing, add washing buffer to every well, still for 30 s then drain, repeat 5 times, dry by pat.
6. Add enzyme: Add HRP-Conjugate reagent 50 μl to each well, except blank well.
7. Incubate: Operation with 3.
8. Washing: Operation with 5.
9. Color: Add Chromogen Solution A 50 μl and Chromogen Solution B to each well, evade the light preservation for 15 min at 37°C
10. Stop the reaction： Add Stop Solution50 μl to each well, Stop the reaction (the blue color change to yellow color).
11. Assay: take blank well as zero, read absorbance at 450nm after Adding Stop Solution and within 15 min.

**3. Calculate**

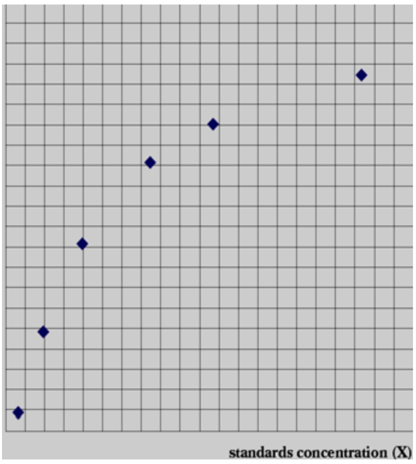


**4. Assay range**

2.5 pg/mL - 80 pg/mL.

**5. Sensitivity**

The minimum detectable dose is typically less than 0.1 pg/mL.

**Immunoglobulin A (IgA) ELISA Kit Instructions (ML002792)**

## 1. Sample collection and storages

Coagulation at room temperature 10-20 mins, centrifugation 20 mins at the speed of 2000-3000 r. p. m. remove supernatant, if precipitation appeared, Centrifugal again.

## 2. Assay procedure

1. Add standard: Set Standard wells, testing sample wells. Add standard 50 µl to standard well.
2. Add sample: Set blank wells separately (blank comparison wells don't add sample and HRP-Conjugate reagent, other each step operation is same). testing sample well. add Sample dilution 40 µl to testing sample well, then add testing sample 10 µl (sample final dilution is 5-fold), add sample to wells, don't touch the well wall as far as possible, and gently mix.
3. Incubate: After closing plate with Closure plate membrane, incubate for 30 min at 37℃.
4. Configurate liquid: 30-fold (or 20-fold) wash solution diluted 30-fold (or 20-fold) with distilled water and reserve.
5. Washing: Uncover Closure plate membrane, discard Liquid, dry by swing, add washing buffer to every well, still for 30 s then drain, repeat 5 times, dry by pat.
6. Add enzyme: Add HRP-Conjugate reagent 50µl to each well, except blank well.
7. Incubate: Operation with 3.
8. Washing: Operation with 5.
9. Color: Add Chromogen Solution A 50 µl and Chromogen Solution B to each well, evade the light preservation for 15 min at 37℃
10. Stop the reaction： Add Stop Solution50 µl to each well, Stop the reaction (the blue color change to yellow color).
11. Assay: take blank well as zero, Read absorbance at 450 nm after Adding Stop Solution and within 15 min.

## 3 Calculate

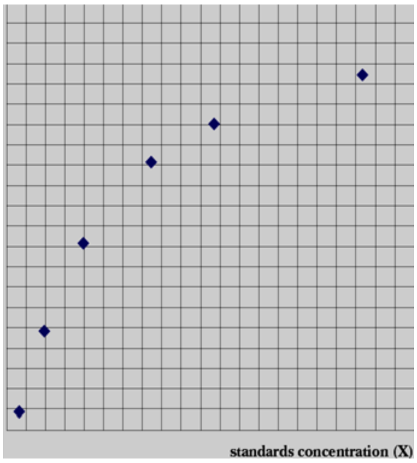


## 4. Assay range

10 µg/mL - 320 µg/mL.

## 5. Sensitivity

The minimum detectable dose is typically less than 1.0 µg/mL.

# File S2

**Antioxidant Indicators Kit Instructions**

| Index | Sampling Volum | Computational Formula | | Kit. No. |
|---|---|---|---|---|
| T-AOC | 10 µl | T-AOC (µmol Trolox/mL) = $[(\triangle A + 0.0218) \div 1.7571 \times V1] \div V1 \times D$ | V1----Volume of sample in reaction, 10 µL=0.01 mL;<br>D----dilution multiple. | G0142W |
| CAT | 10 µl | CAT (µmoL/min/mL) = $[(\Delta A + 0.0025) \div 0.2093] \div V1 \div T = 95.6 \times (\Delta A + 0.0025)$ | V1----volume of sample added, 0.01 mL;<br>T----reaction time, 5 min. | G0105F |
| GSH-Px | 80 µl | GSH-Px (nmol /min/mL) = $[(\triangle A - 0.0008) \div 5.3806 \times 10^3 \times V2] \div V1 \div T \times D$ | V1----supernatliquid product in the reaction system, 80 L =0.08 mL;<br>V2----Total reaction volume at the reaction phase, 1000 µL=1 mL;<br>T----reaction time, 5 min；<br>D----dilution multiple. | G0204F |
| SOD | 20 µl | SOD activity (U/mL) = [inhibition percentage ÷ (1-inhibition percentage) × V2] ÷ V1×D | V1---Volume of sample added to reaction system, 0.02 mL;<br>V2---Total volume of reaction system, 0.2 mL;<br>D---Dilution ratio of sample, undiluted 1; | G0101W |
| MDA | 400 µl | MDA (nmol/mL) = $[\Delta A \div (\varepsilon \times d) \times V2 \times 10^9] \div V1$ | V1---Sample volume of the added reaction system, 0.4 mL;<br>V2---Total reaction liquid product of sample extract and working liquid, $1 \times 10^3$ L;<br>d---The cuvette light diameter, 1 cm;<br>ε---MDA molar extinction coefficient, $155 \times 10^3$ L/mol/cm. | G0109F |

**Total Antioxidant Capacity (T-AOC) Kit Instructions (G0142W)**

## 1. Sample preparation

Liquid samples: direct detection; if turbid, take the supernatant after centrifugation for detection.

## 2. On-board inspection:

① Preheat the microplate reader for more than 30 min and adjust the wavelength to 414 nm.

② Different samples have different removal abilities. Two samples can be selected for testing first. If the A determination-A control is close to zero, the samples need to be diluted (the dilution solution is the same as the tissue extract solution, that is, the water-soluble samples are diluted with PBS or distilled water, The water-insoluble samples were diluted with 80% ethanol) and then tested, and the dilution factor D was substituted into the formula for calculation.

③ Add in order to a 96-well plate:

| Reagent name (μL) | Assay tube | Control tube | Blank tube (do it once) |
|---|---|---|---|
| Sample | 10 | 10 | |
| Diluent to configure working fluid | | 190 | 10 |
| Working fluid | 190 | | 190 |
| Mix well, stand at room temperature (25°C) for 6 min in the dark, read the absorbance value A at 414 nm, △A = A blank - (A measurement - A control). | | | |

## 3. Result calculation:

3.1 Standard curve: y =1.7571x - 0.0218, x is the standard Trolox molar concentration (μmol/mL), y is ΔA.

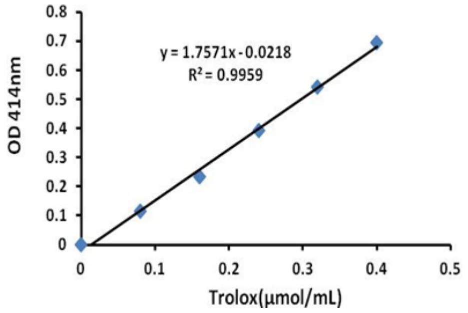


3.2. Calculated by sample mass:

3.3 Liquid sample:

Total antioxidant capacity (μmol Trolox/mL) = [(△A+0.0218) ÷ 1.7571 × V1] ÷ V1 × D = 0.57 × (△A + 0.0218) × D

V----Volume of extracting solution added, 1 mL;

V1----Volume of sample in reaction, 10 μL=0.01 mL;

W----Sample mass, g;

Trolox molecular weight----250.29.

**Catalase (CAT) Kit Instructions (G0105F)**

**1. Sample preparation:**

Liquid samples: direct detection; if turbid, take the supernatant after centrifugation for detection.

**2. Test on the machine:**

① Preheat the spectrophotometer for more than 30 min, adjust the wavelength to 510 nm, and use distilled water to zero.

② Reagent 2 is prepared in advance according to the reagent preparation requirements, and then the following operations are performed.

③ Test the blank tube first **(do it only once)**: 80 μL of reagent 1 + 20 μL of reagent 2 + 100 μL of reagent 3, immediately mix well, take 10 μL, and **immediately** add samples according to the color reaction in step ⑥ for detection. The absorbance value is the A blank.

④ **Suggestion**: Since the reaction time is 5 minutes, if there are many samples to be tested at one time, the samples can be tested in batches.

⑤ Add in sequence to the EP tube:

| Reagent name (μL) | Test tube |
|---|---|
| Sample | 10 |
| Reagent 1 | 70 |
| Reagent 2 | 20 |
| Mix well, (observe the generation of bubbles, the greater the enzyme activity, the more bubbles), and the reaction is carried out **accurately** for 5 minutes at room temperature of 25°C. | |
| Reagent 3 | 100 |
| **Immediately** after mixing, take 10 μL of the mixture | |

⑥ Color reaction:

| Reagent name (μL) | Test tube |
|---|---|
| Mixture | 10 |
| Reagent 1 | 900 |
| Reagent 4 | 290 |
| Mix well, react at room temperature and 25°C for 5min, transfer 1mL to a 1mL glass cuvette, measure the absorbance value A at 510nm, ΔA=A blank - A measure. | |

**3 Result calculation:**

3.1. Standard curve: y = 0.2093x - 0.0025: x is $H_2O_2$ standard (μmoL), y is ΔA.

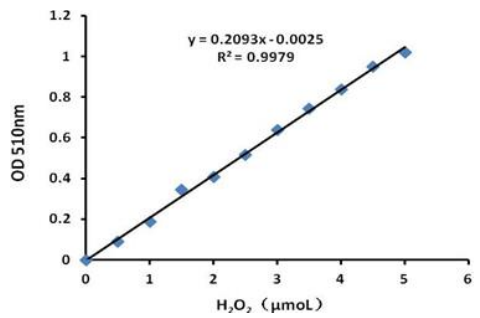


3.2. Calculate according to the liquid volume:

Definition of enzymatic activity: At 25°C, 1 μmoL $H_2O_2$ of catalytic decomposition per milliliter of

liquid per minute is defined as one enzymatic activity unit (U).

$CAT(\mu moL/min/mL) = [(\Delta A + 0.0025) \div 0.2093] \div V1 \div T = 95.6 \times (\Delta A + 0.0025)$

V----volume of extracting solution added, 1 mL;

V1----volume of sample added, 0.01 mL;

T----reaction time, 5 min;

**Glutathione Peroxidase (GSH-Px) Kit Instructions (G0204F)**

**1. Sample preparation:**

liquid sample: direct determination. If cloudy, take the supernatant after centrifugation

**2. Test on the machine:**

① visible spectrophotometer preheated for 30 min, adjusted to 412 nm and adjusted distilled water to zero.

② reagent 1 was preheated one to five in a 25℃ water bath for 30min.

③ Add in EP tube in order:

| Reagent Name (μL) | Determination tube | Blank tube |
|---|---|---|
| Reagent 1 | 80 | 80 |
| sample | 80 | |
| dd water | | 80 |
| Reagent 2 | 40 | 40 |
| Reaction at 25℃ for 5min (strictly controlled time) | | |
| Reagent 3 | 800 | 800 |
| It was centrifuged at 12000 rpm for 10 min and the supernatant was tested | | |

④ color reaction: Add in 1 mL glass cuvette:

| Reagent Name (μL) | Determination tube | Blank tube |
|---|---|---|
| Supernatant of the above steps | 320 | 320 |
| Reagent 4 | 400 | 400 |
| Reagent 5 | 80 | 80 |
| The reaction was 1min and the absorbance value A was read at 412 nm, △A = A blank tube - A measuring tube. | | |

**3. Results calculation:**

Calculate by liquid volume

Activity unit definition: 1 nmol GSH per ml per minute under 25 ℃ reaction conditions.

GSH-Px enzyme was viable(nmol /min/mL) = [(△A - 0.0008) ÷ 5.3806 × 103 × V2] ÷ V1 ÷ T × D = 464.6 × (△A - 0.0008) × D

V----Extract volume, 1 mL;

V1----supernatliquid product in the reaction system, 80 L =0.08 mL;

V2----Total reaction volume at the reaction phase, 1000 μL=1 mL;

D----dilution multiple;

GSH molecular weight-----307.3;

**Superoxide Dismutase (SOD) Kit Instructions (G0101W)**

## 1. Sample preparation:

Liquid samples: direct detection; if turbid, take the supernatant after centrifugation for detection.

## 2. Test on the machine:

① Preheat the microplate reader for more than 30 min and adjust the wavelength to 450 nm.
② Put reagents 1, 3 and 4 in a water bath at 25°C for more than 5 minutes before the measurement.
③ Operate with a row gun to reduce the error caused by the timing of adding reagents between wells.
④ Reagent 4 must be mixed before each sample addition to ensure the uniformity of the reagent.
⑤ Add in order to the 96-well plate:

| Reagent name (μL) | Sample tube | Sample control tube* (optional) | Blank tube 1 **(do it only once)** | Blank tube 2 **(do it only once)** |
|---|---|---|---|---|
| Reagent 1 | 70 | 70 | 70 | 70 |
| Reagent 2 | 20 | | 20 | |
| Distilled water | | 20 | 20 | 40 |
| Sample | 20 | 20 | | |
| Reagent 3 | 10 | 10 | 10 | 10 |
| Reagent 4 | 80 | 80 | 80 | 80 |
| Mix well and stand for 30 min (accurate time) **in the dark at room** temperature (25°C), then measure the absorbance value A of each tube at 450 nm. | | | | |

## 3. Result calculation:

3.1. Calculation of inhibition percentage:
Inhibition percentage = [(A blank tube 1-A blank tube 2) - (A sample tube-A sample control tube*)] / (A blank tube 1-A blank tube 2) × 100%
3.2. SOD enzyme activity calculation:
Calculated by liquid volume:
SOD activity (U/mL) = [inhibition percentage ÷ (1-inhibition percentage) × V2] ÷ V1×D =10 × inhibition percentage ÷ (1 - inhibition percentage) × D
V---Volume of extracting solution added, 1 mL;
V1---Volume of sample added to reaction system, 0.02 mL;
V2---Total volume of reaction system, 0.2 mL;
D---Dilution ratio of sample, undiluted 1;

**Malondialdehyde (MDA) Kit Instructions (G0109F)**

**1. Sample preparation:**

liquid sample: direct detection. If cloudy, take the supernatant after centrifugation.

**2. Check on the machine:**

① open the spectrophotometer for 30 min, distilled water to zero, while heating the water bath to 90-95℃.

② Add in EP tubein order：

| Reagent Name (μL) | Determination tube |
|---|---|
| fluid | 600 |
| Sample | 400 |
| After mixing, kept in 90-95℃ water bath for 30 min, removed and cooled on ice, centrifuged at 12000 rmp for 10min, all the supernatant was transferred in 1 mL glass cuvette, and absorbance A was read at 532 nm and 600 nm, respectively, ΔA = A532 - A600 | |

**3. Results Calculation:**

Volume by liquid:

MDA content (nmol/mL) = $[\Delta A \div (\varepsilon \times d) \times V2 \times 10^9] \div V1 = 16.1 \times \Delta A$

V---The total volume of the sample extract, 1 mL;

V1---Sample volume of the added reaction system, 0.4mL;

V2---Total reaction liquid product of sample extract and working liquid, 1×10-3 L;

d---The cuvette light diameter, 1 cm;

---MDA molar extinction coefficient, 155×10-3 L/mol/cm;
